# The Effect of a Very-Low-Calorie Diet (VLCD) vs. a Moderate Energy Deficit Diet in Obese Women with Polycystic Ovary Syndrome (PCOS)—A Randomised Controlled Trial

**DOI:** 10.3390/nu15183872

**Published:** 2023-09-06

**Authors:** Harshal Deshmukh, Maria Papageorgiou, Liz Wells, Shahzad Akbar, Thomas Strudwick, Ketki Deshmukh, Salvatore Giovanni Vitale, Alan Rigby, Rebecca V. Vince, Marie Reid, Thozhukat Sathyapalan

**Affiliations:** 1 Department of Academic Diabetes and Endocrinology, Allam Diabetes Centre, Hull University Teaching Hospitals NHS Trust, Hull HU3 2JZ, UK; harshal.deshmukh@nhs.net (H.D.); shahzad.akbar1@nhs.net (S.A.); 2 Department of Academic Diabetes and Endocrinology, Hull York Medical School, University of Hull, Hull HU6 7 RX, UK; 3 Faculty of Medicine, University of Geneva, 1205 Geneva, Switzerland; maria.papageorgiou@unige.ch; 4 School of Sport, Exercise and Rehabilitation Sciences, University of Hull, Hull HU6 7 RX, UK; liz.wells@hull.ac.uk (L.W.); rebecca.vince@hull.ac.uk (R.V.V.); 5 School of Psychology and Social Work, University of Hull, Hull HU6 7 RX, UK; thomas.strudwick@eastamb.nhs.uk (T.S.); m.reid@hull.ac.uk (M.R.); 6 Division of Gynecology and Obstetrics, Department of Surgical Sciences, University of Cagliari, 09121 Cagliari, Italy; salvatoreg.vitale@unica.it; 7 Institute of Clinical and Applied Health Research, Hull York Medical School, Hull HU6 7 RX, UK; a.rigby@hull.ac.uk

**Keywords:** PCOS, VLCD, metabolic syndrome, energy deficit diet

## Abstract

We performed an open-label, randomised controlled trial to compare the effects of a very-low-calorie diet (VLCD) vs. moderate energy deficit approach on body weight, body composition, free androgen index (FAI), and metabolic markers in obese women with polycystic ovary syndrome (PCOS). Forty eligible patients were randomly assigned to a VLCD (*n* = 21) or a conventional energy deficit approach (*n* = 19) over the same period. After eight weeks, both groups experienced significant weight loss; however, this was greater in the VLCD arm (−10.9% vs. −3.9%, *p* < 0.0001). There was also a trend towards a reduction in FAI in the VLCD group compared to the energy deficit group (−32.3% vs. −7.7%, *p* = 0.07). In the VLCD arm, two women (18%) had a biochemical remission of PCOS (FAI < 4); this was not the case for any of the participants in the energy deficit arm. There was a significant within-group increase in the sex-hormone-binding globulin (*p* = 0.002) and reductions in fasting blood glucose (*p* = 0.010) and waist to hip ratio (*p* = 0.04) in the VLCD arm, but not in the energy deficit arm. The VLCD resulted in significantly greater weight reduction and was accompanied by more pronounced improvements in hyperandrogenaemia, body composition, and several metabolic parameters in obese women with PCOS as compared to the energy deficit approach.

## 1. Introduction

Polycystic ovary syndrome (PCOS) is the most prevalent endocrine disorder, affecting 5–21% of reproductive-aged women [1,2]. These prevalence rates are reported to range depending on the definition employed and the population under investigation [1,2]. Insulin resistance (IR) and resulting hyperandrogenism are cardinal features of PCOS contributing to clinical symptoms, including hirsutism, acne, and polycystic ovary morphology on ultrasound [3]. PCOS is recognised as a leading cause of anovulatory infertility, while in the case of pregnancy, it increases the risk of associated complications [3,4]. In addition to these unfavourable reproductive consequences, women with PCOS are at greater risk of metabolic disorders, including type 2 diabetes mellitus (T2DM), metabolic syndrome, and cardiovascular disease. They are also more likely to experience compromised psychological wellbeing, as evidenced by a high prevalence of anxiety, depression, and body dissatisfaction alongside the lower quality of life reported in this population [5,6,7].

Many women with PCOS experience difficulties in maintaining healthy body weight. Indeed, previous research has shown up to 75% of women with PCOS are overweight/obese [8], whilst affected women may experience increased weight gain longitudinally [9]. Obesity and, in particular, central type obesity, appear to exacerbate IR, hyperandrogenism, reproductive disturbances, and cardiovascular risk factors and intensify psychological consequences [9]. Conversely, weight loss has beneficial effects on PCOS-related outcomes. Lifestyle modifications (diet, physical activity, and behavioural changes) and weight management are recommended as first-line therapy for PCOS to enhance hormonal abnormalities and fertility and prevent long-term metabolic complications [3]. Lifestyle interventions and weight loss are also recommended before conception and initiation of infertility treatments [3] and may lead to higher ovulation rates than treatment with oral contraceptives [10]. Recent data also suggest improvements in psychological outcomes after weight loss in PCOS [11].

Studies involving dietary energy restriction for weight loss in PCOS have mainly focused on moderate reductions in energy intake to induce a deficit of 500–1000 kcal/d with/without the use of anti-obesity/anti-diabetes medication [12,13,14,15,16], whilst the effects of very-low-calorie diets (VLCDs) remain understudied in this population [17,18,19]. VLCDs are defined as dietary plans that provide ≤800 kcal/d. They typically involve partial or complete replacement of meals with synthetic formulas (e.g., shakes, soups, or bars), which are commonly nutritionally replete (i.e., sufficient amounts of vitamins and minerals) to meet dietary requirements. Although VLCDs are recommended for short periods (8–16 weeks) and under medical supervision due to their extreme caloric restriction, they can result in rapid weight loss (20–30%) and, potentially, weight loss maintenance [20]. A growing body of evidence advocates the use of VLCDs in adults with T2DM, as adherence to this type of diet has been shown to augment insulin secretion from the pancreas and reduce HbA1c levels to pre-diabetic and normal levels, thus reversing T2DM [21]. Women living with PCOS have a similar metabolic profile to patients with T2DM [22], and thus, VLCDs may be an attractive, yet underexplored, option in this population.

Thus, the present study aimed to compare the effects of a VLCD vs. a conventional energy deficit approach on body weight and body composition, androgen levels, and other hormonal and metabolic parameters in overweight/obese women with PCOS.

## 2. Materials and Methods

### 2.1. Study Objectives and Design

The primary study objective was to assess the effects of VLCD and conventional energy deficit diet on change in free androgen index (FAI), while the secondary study objective was to assess the effect of both diets on changes in weight, waist circumference, body composition, and other metabolic parameters.

This open-label, randomised, comparative study in women with PCOS was performed in the Academic Diabetes, Endocrinology and Metabolism research centre at Hull Royal Infirmary. Participants were included if they were women wishing to lose weight, aged between 18 and 45 years, had a body mass index (BMI) between 30 and ≤45 kg/m^2^ (based on the dimensions of the DEXA scanner), and were diagnosed with PCOS based on the Rotterdam criteria (biochemical hyperandrogenism, as indicated by a FAI > 4, and self-reported oligomenorrhoea (cycle length > 35 days and nine or fewer periods per year) or amenorrhoea (absence of menses for a period ≥ 3 months)). To be included, participants must have been willing to use a reliable form of non-hormonal contraception throughout the duration of the study. Women with differential diagnoses of non-classical 21-hydroxylase deficiency, hyperprolactinaemia, Cushing’s disease, and androgen-secreting tumours were excluded from participation. Additional exclusion criteria included menopause and perimenopause, pregnancy or intention to become pregnant, breastfeeding, weight loss > 5 kg within the last 6 months, substance abuse, acute illness, diagnosis of diabetes, history or presence of malignant neoplasms within the last 5 years, history of gallstones/gout, inadequately controlled thyroid disorder, diagnosis of eating disorder or purging in the last 12 months (based on patient reporting, results of Eating Disorder Inventory, 3 Referral Form (EDI-3 RF) interpreted by a clinical psychologist), known intolerance to the ingredients of investigational products used in the study (e.g., soy, lactose, gluten), or coeliac disease. Participants who were using the following drugs (within the last three months) were also excluded from participation unless cessation of the drug was agreed upon between the medical team and the patient and a wash-out period of 4–8 weeks was achieved; these drugs were: oral hormonal contraceptives and hormone-releasing implants, anti-androgen (e.g., spironolactone, flutamide, finasteride), metformin or other insulin-sensitising medications, clomiphene citrate or estrogen modulators, gonadotropin-releasing hormone (GnRH) modulators (e.g., leuprolide), Minoxidil, anti-obesity drugs, or other medication that may affect appetite (e.g., oral steroids). All participants provided their written informed consent. Ethical approval has been granted by Yorkshire and Humber—Sheffield Research Ethics Committee, NHS, HRA (17/2/17 REC–16/YH/0518).

Participants attended an initial visit (Visit 1), during which they were screened against inclusion and exclusion criteria by medical history and clinical examination, routine blood tests (i.e., full blood count, liver function tests, urea and electrolytes, clotting screen), and anthropometric measurements. To screen out eating disorders and provide a baseline level for anxiety and depression, participants completed an eating disorder questionnaire, EDI-3 RF, and the Beck inventory questionnaires to assess levels of anxiety and depression. At screening, these were assessed by a psychologist, and a decision was made as to whether they should be excluded. Eligible participants were randomly assigned either a VLCD or a conventional approach of moderate energy deficit for 16 weeks (8 weeks intervention and 8 weeks of diet reintroduction and follow-up). The allocation was generated using an individual that was independent of the study team (unit manager), to ensure that the allocation was truly random and unbiased. Eligible participants were randomised on a 1:1 ratio using an online web-based randomisation service (https://www.randomizer.org/ (accessed on 1 January 2017)).

During Visit 2 (baseline), conducted within 4 weeks of Visit 1, participants underwent anthropometric evaluation (weight, BMI, waist circumference (WC), and hip circumference (HC)) and an evaluation of body composition. They also had blood samples taken (fasting glucose, fasting insulin, HOMA-IR, total cholesterol, low-density lipoprotein cholesterol (LDL-C), high-density lipoprotein cholesterol (HDL-C), triglycerides (TG), and high-sensitivity C-reactive protein (hs-CRP)), and their blood pressure measured. For the first eight weeks, the VLCD group was instructed to follow a prescription of 800 kcal a day (irrespective of their baseline body weight), in the form of soups and drinks made from pre-prepared sachets provided by the Cambridge Weight Plan™ company (Corby, UK). Each meal replacement drink provided 200 kcal, 21 g CHO, 15 g of protein, 3–4 g fats, and was nutritionally complete for micronutrients as they are specifically designed to be used as a sole source of nutrition and total meal replacement diets (The Cambridge Weight Plan Ltd., (Corby, UK)). Participants in this group were provided support and information regarding the consumption of the food replacement sachets, fibre supplement prescription, and fluid consumption. After these first eight weeks, participants in the VLCD arm were given a stepped return, an increase of 200 kcal/2 weeks whilst reducing meal replacement drinks until ~1600 kcal/d was reached. The energy deficit approach group acted as the control group in this trial. The kcal prescription was bespoke for each patient and calculated using the Henry equation based on gender, age, and weight to ascertain basal metabolic rate [23], which was then multiplied by physical activity level (PAL). Once the patients’ daily kcal requirements had been calculated, a deficit of 600 kcal from requirements was applied [24]. Both groups received dietetic support and education on different aspects including portion sizes and kcals, practical measures to achieve the given energy prescription, and healthy eating practices based on the “Eat Well Guidelines” (https://www.nhs.uk/live-well/eat-well/food-guidelines-and-food-labels/the-eatwell-guide/ (accessed on 4 January 2017)). Participants returned for review two weeks after commencement on the VLCD and conventional energy deficit approach (Visit 3), and thereafter support was provided every two weeks via face-to-face or telephonic consultation (Visits 4, 5, 7, 8, and 9). For the purposes of these visits, participants were asked to complete a 3-day food and mood diary (2 weekdays and 1 weekend day), which was reviewed by research staff at each review appointment. Specifically, this review visit included an assessment of bowel habits, dietary intake, compliance, level of motivation, support/education, and encouragement. The details of data collected at each visit are given in Supplementary Table S1. The decision to use a diary for only 3 days (2 weekdays and 1 weekend day) instead of a full diary was based on practical considerations related to the study design and participant burden.

The primary study results presented are from Visit 6, corresponding to the 8-week follow-up, during which all the measurements performed during Visit 2 (baseline) were repeated.

### 2.2. Procedures

Height and weight were recorded with participants wearing light clothing and no shoes using a stadiometer and a weighing scale (MS-4202L, Marsden Weighing Machine Group Limited, Rotherham, UK). BMI was calculated as weight (kg) divided by the square of height (m^2^). Blood pressure was measured using an automated device (NPB-3900; Nellcor Puritan Bennett, Pleasanton, CA, USA); for this measurement, subjects were seated quietly for at least 5 min and with the right arm supported at heart level. Three readings were taken, each at least 2 min apart, and then the mean value of the readings was calculated. Waist circumference was measured using a tape measure. The tape measure was wrapped around participants’ waist at the midway point between the bottom of the ribs and the top of the hips (iliac crest). The participants were encouraged to breathe naturally during the procedure, relax their abdominal muscles and not hold their breath. Body composition including total fat and trunk mass, lean body mass (LBM), fat-free mass, bone mineral content (BMC), and bone mineral density (BMD) were measured at baseline and follow-up visits by dual-energy X-ray absorptiometry (DEXA). Oral glucose tolerance was performed after an overnight fast using a 75 g glucose load.

### 2.3. Biochemical Analysis

Venous blood samples were collected in the fasting state after an overnight fast and after 2 h OGTT at baseline and at 8 weeks. Serum and plasma samples were separated by centrifugation at 2000× *g* for 15 min at 4 °C, and the aliquots were sent immediately for routine biochemical analysis or stored at −80 °C until batch analysis. Serum insulin was assayed using chemiluminescent immunoassay on the Beckman Coulter UniCel DxI 800 analyser (Beckman Coulter UK Ltd., High Wycombe, UK). Plasma glucose was measured using a Beckman AU 5800 analyser (Beckman-Coulter, High Wycombe, UK) according to the manufacturer’s recommended protocol. Insulin resistance was computed using homeostatic model assessment-insulin resistance (HOMA-IR = (fasting serum insulin (µU/mL) × fasting plasma glucose (mmol/L))/22.5). Serum testosterone was quantified using isotope-dilution liquid chromatography tandem mass spectrometry (LC-MS/MS). Sex-hormone-binding globulin (SHBG) was measured using a chemiluminescent immunoassay on the UniCel DxI 800 analyser (Beckman-Coulter, High Wycombe, UK), applying the manufacturer’s recommended protocol. The FAI was calculated as: (total testosterone/SHBG) × 100. An FAI of ≥4 was considered as significant hyperandrogenaemia, and <4 at follow-up was considered as biochemical remission of PCOS. Free testosterone and FAI are effective in detecting elevated androgen levels. In women, a significant amount of testosterone is bound to SHBG, making the interpretation of free testosterone levels more difficult. The FAI compensates for this dependence on SHBG by taking it into account. There are no universally accepted definitions for biochemical remission of PCOS; however, FAI levels of 5 or higher are considered indicative of PCOS [25,26]. The cut-off value of FAI for diagnosis of PCOS is lab specific, and in our hospital, a FAI of more than 4 is regarded as indicative of PCOS. Hence, we defined FAI < 4 as biochemical remission of PCOS. Total cholesterol, triglycerides, HDL-C, alanine aminotransferase (ALT), and aspartate aminotransferase (AST) levels were measured enzymatically using a Beckman AU 5800 analyser (Beckman-Coulter, High Wycombe, UK). Low-density lipoprotein cholesterol (LDL-C) was calculated using the Friedwald equation.

### 2.4. Statistical Analysis

The continuous variables in the study were summarised as means ± SD, while the categorical data are presented as *n* (%). Mean changes from baseline to 8-week follow-up within each treatment group were analysed using a paired *t*-test. Mean differences for all parameters expressed as % change from baseline between groups were analysed using independent samples *t*-tests. The power of this study was nominally based on a correlation between weight loss and free-androgen reduction. Ten patients completing will allow us to detect a correlation of 0.75 (power = 0.80, alpha = 0.1). Alpha was one-tailed, since we were only interested in a one-directional change. All the statistical analyses were performed in R4.1.1 (https://www.r-project.org/ (accessed on 1 January 2021)) with *p*-values of less than 0.05 denoting statistical significance.

## 3. Results

**Figure 1:**
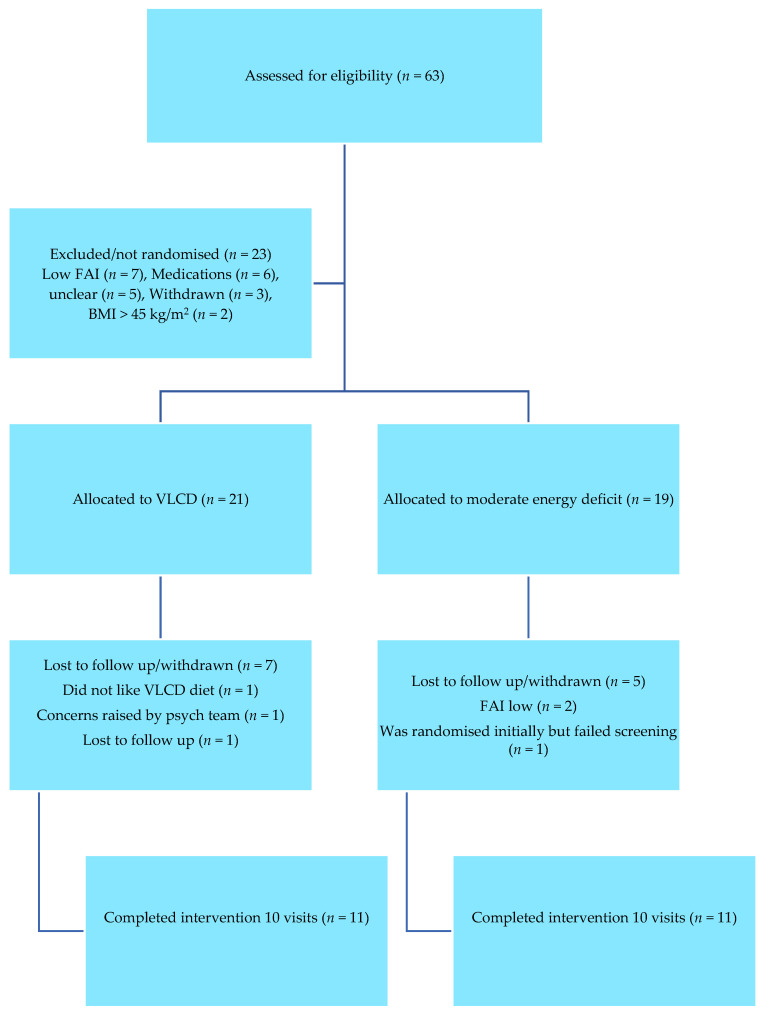
Consort flow diagram of participants.

Figure 1 shows the study Consort diagram. We screened 63 women living with PCOS, out of which 23 were not randomised due to not meeting the eligibility criterion. Overall, 21 women were randomised to the VLCD arm, and 19 women living with PCOS were randomised to a conventional energy deficit diet. Subsequently, 11 participants in the VLCD arm and 11 participants in the conventional energy deficit arm completed the 8-week follow-up. Table 1 shows the demographic characteristics of the study population.

### 3.1. Effect on Free Androgen Index (FAI)

**Figure 2:**
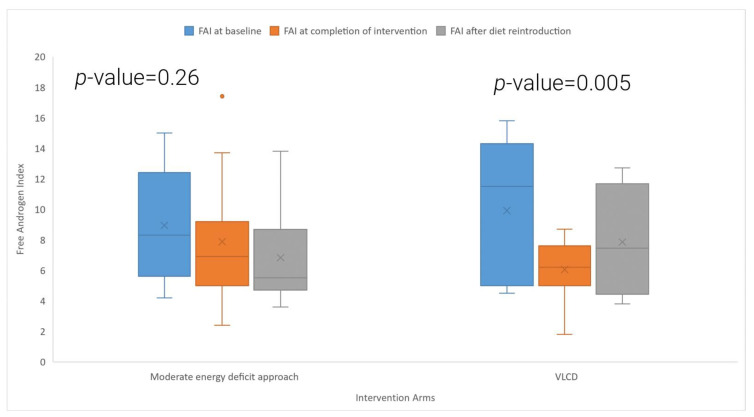
Comparison of baseline FAI, FAI at completion of intervention, and after the diet reintroduction in the VLCD and moderate energy deficit approach.

Table 2 and Figure 2 shows the effects of the VLCD and the conventional energy deficit approach on FAI. In the VLCD arm, there was a statistically significant reduction in the FAI at the 8-week follow-up (−32.3% change from baseline levels; baseline: 9.9 ± 4.3, 8-week follow-up: 6.1 ± 1.9, *p* = 0.005), while the conventional energy deficit group experienced a 7.7% reduction in FAI, which was, however, not statistically significant (*p* = 0.26). Between-group comparisons of the mean % reductions from baseline in FAI showed a trend towards a greater reduction In the VLCD group (*p* = 0.07). In the VLCD arm, 36% (4 out of 11) participants had more than 50% reduction in FAI, and 73% (8 out of 11) of the participants had more than 20% reduction in FAI at the end of the 8-week period. In the conventional energy deficit arm, 9% (1 out of 11) of the participants had more than 50% reduction in FAI, and 36% (4 out of 11) of the participants had more than 20% reduction in FAI at the 8-week follow-up. Two women in the VLCD arm (18%), but none in the conventional energy deficit arm, had a biochemical remission of PCOS (FAI < 4). Across both study arms, there was a significant correlation between weight loss and reductions in FAI (r^2^ = 0.51 and *p* = 0.01).

Figure 2 shows the box and whisker plot comparing free androgen index (FAI) at baseline, after the intervention and after the reintroduction of the diet in the two study arms. The symbol “X” in the box and whisker plot shows the mean value, and the orange dot shows a value 1.5 times the interquartile range above the upper or lower quartile.

The *p*-value is derived from a *t*-test comparing baseline FAI and FAI at eight weeks’ follow-up in the moderate energy deficit arm and VLCD arm.

### 3.2. Body Weight and Waist Circumference

**Figure 3:**
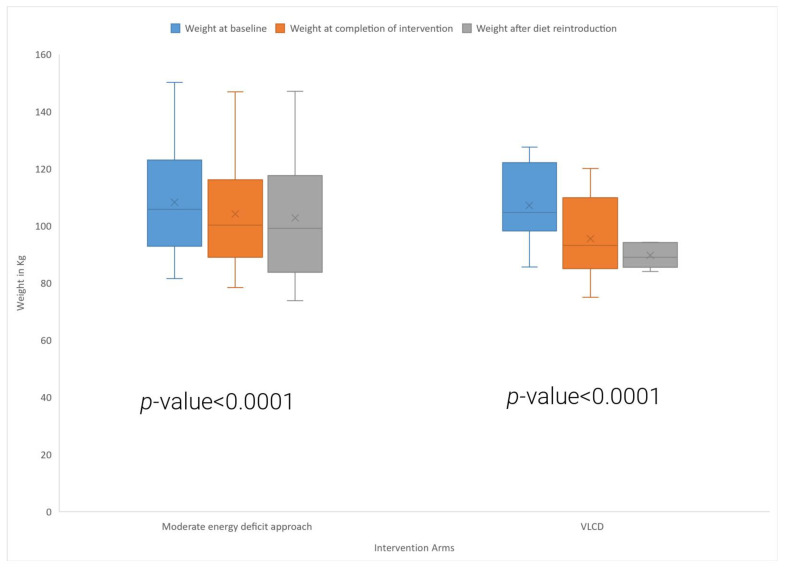
Comparison of baseline weight, weight at completion of intervention, and after the diet reintroduction in the VLCD and moderate energy deficit approach.

**Figure 4:**
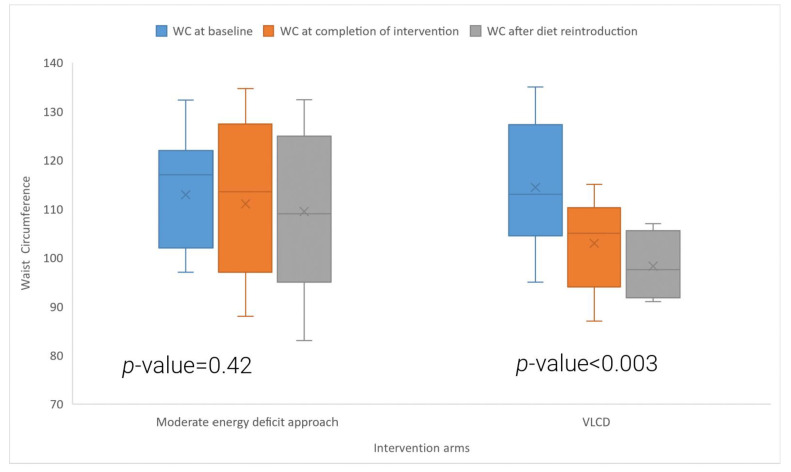
Comparison of baseline waist circumference (WC), WC at completion of intervention, and after the diet reintroduction in the VLCD and moderate energy deficit approach.

Table 2 and Figure 3 and Figure 4 show the effects of the VLCD and conventional energy deficit approach on body weight and waist circumference in women living with PCOS. Participants in the VLCD arm experienced a significant 10.9% reduction in their body weight after 8 weeks of VLCD (baseline: 107.1 ± 13.6 kg, 8-week follow-up: 95.4 ± 13.2 kg, *p* < 0.0001). Participants who followed the conventional energy deficit approach also had a significant reduction in their body weight and had a 3.9% reduction in their body weight (baseline: 108.3 ± 20.5 kg, 8-week follow-up: 104.1 ± 20.6 kg, *p* < 0.0001). Comparisons between groups revealed significantly greater weight loss in the VLCD group compared to the conventional energy deficit group (*p* < 0.0001). There was a significant reduction in waist circumference in the VLCD arm (Figure 3) (baseline: 114.4 ± 12.6 cm, 8-week follow-up: 102.9 ± 9.1 cm, *p* = 0.003), but not in the conventional energy deficit arm. In the VLCD arm, all the participants lost >5% of their body weight, with seven participants losing >10% of body weight; in the energy deficit arm, four participants lost >5% of body weight, and none of them lost >10% body weight.

Figure 3 shows the box and whisker plot comparing weight at baseline, after the intervention, and after the reintroduction of the diet in the two study arms. The symbol “X” in the box and whisker plot shows the mean value.

The *p*-value is derived from a *t*-test comparing baseline weight and weight at eight weeks’ follow-up in the moderate energy deficit arm and VLCD arm.

Figure 4 shows the box and whisker plot comparing WC at baseline, after the intervention, and after the reintroduction of the diet in the two study arms. The symbol “X” in the box and whisker plot shows the mean value.

The *p*-value is derived from a *t*-test comparing baseline WC and WC at eight weeks follow-up in the moderate energy deficit arm and VLCD arm.

### 3.3. Metabolic Parameters

In the VLCD group, there was a significant increase in the SHBG levels (*p* = 0.002) and significant reductions in total cholesterol (*p* = 0.01) and fasting blood glucose (*p* = 0.01) levels after eight weeks of intervention, however, there were no significant changes in 2 h glucose levels after an OGTT, nor to HBA1c or TG levels. Total cholesterol levels were also reduced in the energy deficit group (*p* = 0.01); however, no further significant changes were seen for other metabolic parameters within the same timeframe. The increase in SHBG (*p* = 0.02) and the reduction in fasting blood glucose levels (*p* = 0.04) were significantly larger in the VLCD arm as compared to the conventional energy deficit arm. There was also a significant reduction in HOMA-IR in both the VLCD (*p* = 0.0007) arm and conventional energy deficit group (*p* = 0.009) but no significant difference between the two arms (*p* = 0.24).

### 3.4. Parameters of Body Composition

There were significant reductions in total and trunk fat in both study arms (Table 3); however, these were more pronounced in the VLCD group as compared to the conventional energy deficit group (total body fat: −15.8% vs. −4.9%, *p* < 0.0001; trunk fat: 17.3% vs. −5.2% *p* < 0.0001). Both diets were associated with significant reductions in LBM and fat-free body mass (FFM) (*p* < 0.05), although these changes were smaller in the conventional energy deficit group (LBM, *p* = 0.002; FFM, *p* = 0.001). There were no significant changes in BMC or BMD in either study arm.

### 3.5. FAI and Weight at 16-Week Follow-Up

Between 8 and 16 weeks (end of the VLCD diet and reintroduction of a normal diet), two participants were lost to follow-up, and four participants did not have their FAI and weight measured in the VLCD arm. One participant withdrew from the study in the energy deficit diet arm over the same period. At the end of the 16-week period, participants in the VLCD arm had statistically significantly more weight loss than the energy deficit arm (−14.3% vs. −6.4% *p* = 0.0001); however, there were no significant differences in FAI changes (−15.9% vs. −19.6% *p* = 0.79) between the two groups.

### 3.6. Side Effects in Study Arms

The most prevalent side effect in both the study arms was gastrointestinal disturbances. The majority of the study participants experienced transient constipation, bloating, and minor abdominal discomfort at some point during the eight weeks of the VLCD or energy deficit arm; however, these were resolved after prescribing a fibre supplement and/or providing advice for fluid intake. One of the study participants in the VLCD arm was admitted to the hospital with acute cholecystitis and had an uneventful recovery. No other major side effects were reported during the trial period.

## 4. Discussion

In this first randomised controlled trial looking into the effects of a VLCD compared to a conventional energy deficit approach on PCOS-related outcomes, we showed that although both strategies can induce short-term weight loss with favourable changes in body composition, implementation of a VLCD resulted in greater weight loss and more pronounced improvements in body composition, hyperandrogenaemia, and metabolic parameters in obese women with PCOS.

Excess weight is an independent risk factor for hyperandrogenaemia, insulin resistance, and menstrual irregularities in women living with PCOS. Weight loss with lifestyle and dietary changes is the mainstay of management of women with PCOS, and it is associated with significant improvements in hyperandrogenaemia, menstrual irregularities, ovulation, and emotional wellbeing in this population [3]. Several dietary interventions have been proposed for the management of PCOS, including VLCDs [18,19,27], energy deficit diets [28,29], and low-GI [30] and ketogenic diets [31,32]. There is, however, no consensus on dietary interventions for optimal weight loss strategies [33]. Recent data from people living with T2DM have shown up to 15% weight loss with VLCDs over twelve weeks in this population [21], which was sustained in over one-third of the study participants at the end of two years. Since the insulin resistance state in people living with T2DM is also seen in women living with PCOS [22], this diet is an attractive option for weight loss in PCOS.

In this study, we presented both within-group and between-group analyses. Although the within-group analysis in a setting for RCT could be biased due to small sample sizes [34], the effectiveness of VLCDs in T2DM is established, and we wanted to compare its effects in the PCOS population. Indeed, in our study, we showed that participants in the VLCD arm lost on average ~11% of their initial body weight after 8 weeks of following a VLCD, while some participants who successfully completed the 16-week follow-up had a mean weight loss of 16%. Participants in the conventional energy deficit group experienced a significant, albeit modest, weight loss (−3.9% from baseline at 8 weeks), suggesting that the use of VLCDs is superior for weight loss in this population, at least in the short term. Our results are in line with previous research [17,35] investigating the effect of VLCDs in women living with PCOS. A mean weight loss of 17% was reported in a retrospective analysis [35] of a 12-week community-based dietary intervention (LighterLife Total (LLT)) consisting of a commercial VLCD in combination with group behavioural change sessions in women with PCOS. Interestingly, this study included a control group of women without PCOS, who experienced a similar weight loss after the use of a VLCD over the same period. In another study involving the implementation of an energy-restricted diet providing 1000 kcal/day, Tolino et al. [17] reported that 54% of the participants had a ≥5% reduction of their baseline weight; nevertheless, no control group was available. Moran et al. [9], tested the efficacy of partial meal replacement with commercial products as part of an energy-restricted diet, and participants reduced their body weight by 5.6 ± 2.4 kg after an 8-week period. Notably, their energy restrictions were more moderate (1000–1200 kcal/d) compared to the energy prescription in our VLCD group (800 kcal/d based on full meal replacement). We reported a slightly lower weight loss with the VLCD in our study as compared to other T2DM cohorts [21]; this could be due to the difference in the mechanism of insulin resistance in women with PCOS as compared to T2DM, and further studies are needed to confirm this effect.

There are very limited data on the effects of VLCDs on hyperandrogenaemia and metabolic outcomes in women with PCOS [18,19,27,36]. In this RCT, we showed that a VLCD caused a significant mean 32% reduction in FAI in the VLCD arm and a non-significant 8% reduction in the conventional energy deficit arm. An observational study [36] compared three months of weight reduction by using a VLCD with the use of an oral contraceptive (OC) pill containing norethisterone in women living with PCOS. This study showed a significant reduction in free testosterone in both the VLCD and OC arm; however, as expected, no significant reductions in body weight or BMI were observed in the OC arm. In the study of Tolino et al., caloric restriction (1000 kcal/d) for four weeks resulted in an increase in SHBG levels and decreases in free testosterone and insulin, with consequent improvement in symptoms of PCOS. Taken together, these findings suggest that a VLCD can be used as an effective management strategy in women living with PCOS who require both amelioration of symptoms of excess androgens and weight reduction.

In this study, we showed that the significant weight loss after eight weeks after both interventions was due to improvements in total and trunk fat masses but also due to reductions in FFM, with these results being in line with earlier studies in PCOS and non-PCOS populations [19]. These changes were greater in magnitude in the VLCD group and can be explained by the greater weight loss experienced by this group. There is increasing evidence that hyperandrogenism in women with PCOS contributes to insulin resistance and metabolic dysfunction in women with PCOS by favouring abdominal and visceral adiposity [37]. The improvement in hyperandrogenism and central obesity mediated by VLCD can improve metabolic dysfunction and associated complications in women with PCOS. Indeed, in parallel with weight loss, reductions in body fat, and hyperandrogenaemia, we observed favourable changes in metabolic markers, including decreases in total cholesterol and fasting blood glucose, in particular in the VLCD group.

This study shows that both VLCD and energy deficit diets can be effectively used in women with PCOS to achieve weight loss with relatively few side effects. One of the study participants in the VLCD had abdominal pain, was diagnosed with cholecystitis, and recovered completely. Some participants had some gastrointestinal side effects, such as constipation and bloating, after starting the VLCD; they were prescribed a fibre supplement to resolve these symptoms, and none of them withdrew from the study due to this reason. It should also be noted that there was a rise in AST and ALT levels in the VLCD arm, which, however, did not reach significance. Increases in hepatic enzymes are a known consequence of rapid weight loss [38], and given the high prevalence of non-alcoholic fatty liver disease in women with PCOS [39], careful monitoring of liver function is warranted while using VLCD. Neither the VLCD nor the conventional energy deficit approach was associated with significant reductions in BMC or BMD in this study. There are conflicting data [40,41,42,43] in the literature on the effect of short-term weight loss on BMC and BMD, and future studies are needed to confirm these findings using longer-term protocols.

Our study had several limitations. This study was a small, single-centre trial, and thus, future multicentre trials will be needed to further understand the feasibility and efficacy of VLCDs in PCOS. Furthermore, half of the study participants did not complete the clinical trial after randomisation in both study arms. Similar to our study, some previous weight loss studies in PCOS have also reported low completion rates, whilst qualitative studies have shed light on a number of barriers to weight management in this population. In our study, several participants randomised to the conventional energy deficit approach were disappointed by their randomisation order, and some of them were lost to follow-up a few weeks after the initiation of the intervention despite the repeated efforts of the research team to keep them in the study and explain to them the benefits of weight loss and the advantages of the conventional energy deficit approach. In the VLCD arm, many participants limited their engagement with the trial in the food reintroduction period. This limited engagement could be because the main study dietician who was in constant touch with the study participants had moved away in the later stage of the trial and highlights the importance of the interpersonal touch during dietary intervention studies in young populations. It is also possible that the participants did not feel the need to engage with the trial protocols after losing a considerable amount of weight. Other challenges mentioned by the participants over the follow-up were fatigue with sticking to study regimens, challenges in keeping up with social life and family issues, and commitment for follow-up. Finally, the last follow-up after the end of the intervention (eight weeks) was at twelve weeks, and the sustainability of weight loss with these two approaches beyond this period will have to be examined with studies with longer follow-up periods.

## 5. Conclusions

In summary, the results of this randomised controlled trial comparing a VLCD based on full meal replacement with a conventional energy deficit approach in PCOS suggests that both approaches can be used to achieve short-term weight loss in this population. However, the study found that the VLCD resulted in greater weight loss and more pronounced improvements in body composition, hyperandrogenism, and metabolic aspects related to PCOS. While these findings are promising, these are based on a small, single-centre study, and further large multicentre RCTs are needed to evaluate the widespread use of VLCDs and moderately energy-restricted diets for managing overweight/obesity in women with PCOS.

## Figures and Tables

**Table 1:** Baseline characteristics of the study population.

	VLCD Arm (*n* = 11)	Moderate Energy Deficit Arm (*n* = 11)
Age (years)	27.7 (±3.8)	28.1 (±5.6)
Weight (kg)	107.09 (±13.6)	108.25 (±20.5)
BMI (kg/m^2^)	37.8 (±3.9)	37.6 (±5.07)
WC (cm)	114.4 (±12.6)	112.9 (±11.7)
WHR	0.92 (±0.1)	0.88 (±0.05)
FAI	9.9 (±4.3)	8.9 (±7.8)
DHEAS (µmol/L)	6.9 (±3.3)	6.6 (±4.5)
Androstenedione (nmol/L)	5.3 (±2.0)	5.8 (±1.8)
SHBG (nmol/L)	16.0 (±6.5)	20.4 (±3.5)
LH (iu/L)	8.2 (±4.3)	9.6 (±5.2)
FSH (iu/L)	6.9 (±1.7)	5.8 (±1.0)
Fasting BS (mmol/L)	4.9 (±0.4)	4.6 (±0.2)
2-h post meal BS (mmol/L)	5.8 (±1.2)	5.1 (±1.1)
HbA1c (mmol/mol)	36.2 (±1.6)	37.0 (±3.7)
Total Cholesterol (mmol/L)	4.9 (±0.7)	4.6 (±0.6)
TG (mmol/L)	1.6 (±0.7)	1.2 (±0.5)
CRP (mg/L)	7.0 (±5.7)	6.8 (±5.1)
ALT (iu/L)	25.3 (±10.8)	29.8 (±13.3)
AST (iu/L)	19.7 (±4.2)	22.6 (±5.2)

BMI = body mass index; WC = waist circumference; WHR = waist/hip ratio; FAI = free androgen index; DHEAS = dehydroepiandrosterone sulphate; SHBG = sex-hormone-binding globulin; LH = luteinizing hormone; FSH = follicle-stimulating hormone; BS = blood sugar; TG = triglycerides; CRP = C-reactive protein; ALT = alanine aminotransferase; AST = aspartate aminotransferase. Results are expressed as mean (±SD) or percentages.

**Table 2:** Changes in metabolic and hormonal parameters in women with PCOS with VLCD and moderate energy deficit at 8-week follow-up.

	VLCD Arm (*n* = 11)				Moderate Energy Deficit Arm (*n* = 11)				
	Baseline	Follow-Up	Mean % Change	*p*-Value ^1^	Baseline	Follow-Up	Mean % Change	*p*-Value ^1^	*p*-Value ^2^
FAI	9.9 (4.3)	6.05 (1.9)	−32.30	**0.005**	8.9 (7.8)	7.8 (4.2)	−7.70	0.260	0.070
Total testosterone (nmol/L)	1.50 (0.70)	1.39 (0.65)	−7.33	0.11	1.81 (0.78)	1.60 (0.78)	−11.6	0.21	0.76
DHEAS (µmol/L)	6.9 (3.3)	7.8 (3.4)	10.30	0.440	6.6 (4.5)	6.5 (4.9)	−6.90	0.160	0.960
Androstenedione (nmol/L)	5.3 (2.0)	4.7 (1.7)	−1.70	0.320	5.8 1.8)	5.05 (2.1)	−11.50	0.140	0.070
Weight (kg)	107.09 (13.6)	95.4 (13.2)	−10.90	**<0.0001**	108.25 (20.5)	104.1 (20.6)	−3.90	**<0.0001**	**<0.0001**
BMI (kg/m^2^)	37.8 (3.9)	33.7 (3.6)	−10.80	**<0.0001**	37.6 (5.07)	35.9 (5.1)	−4.50	**<0.0001**	**<0.0001**
WC (cm)	114.4 (12.6)	102.9 (9.1)	−11.60	**0.003**	112.9 (11.7)	111.03 (15.6)	−1.80	0.420	**0.001**
WHR	0.92 (0.1)	0.86 (0.08)	−11.70	**0.040**	0.88 (0.05)	0.91 (0.07)	−1.86	0.300	**0.001**
SHBG (nmol/L)	16.0 (6.5)	22.8 (7.7)	48.30	**0.002**	20.4 (3.5)	21.9 (8.2)	6.80	0.520	**0.020**
LH (iu/L)	8.2 (4.3)	8.5 (4.1)	14.60	0.090	9.6 (5.2)	8.4 (4.9)	23.60	0.440	0.820
FSH (iu/L)	6.9 (1.7)	6.3 (1.7)	−1.56	0.620	5.8 (1.0)	5.6 (1.3)	−1.71	0.650	0.980
Fasting blood glucose (mmol/L)	4.9 (0.4)	4.4 (0.3)	−7.97	**0.010**	4.6 (0.2)	4.7 (0.3)	−0.02	0.930	**0.040**
2 h blood glucose OGTT (mmol/L)	5.8 (1.2)	6.0 (1.1)	2.10	0.700	5.1 (1.1)	5.9 (1.1)	19.40	0.140	0.200
HbA1c (mmol/mol)	36.2 (1.6)	35.0 (1.7)	−3.10	0.080	37.0 (3.7)	36.4 (4.2)	−1.70	0.190	0.520
Total cholesterol (mmol/L)	4.9 (0.7)	4.1 (0.7)	−13.30	**0.010**	4.6 (0.6)	4.2 (0.6)	−9.10	**0.010**	0.440
TG (mmol/L)	1.6 (0.7)	1.6 (0.4)	−14.20	0.090	1.2 (0.5)	1.2 (0.5)	−1.85	0.430	0.270
CRP (mg/L)	7.0 (5.7)	6.3 (5.5)	6.60	0.760	6.8 (5.1)	6.1 (5.6)	−16.60	0.330	0.280
ALT (iu/L)	25.3 (10.8)	42.6 (28.1)	96%	0.180	29.8 (13.3)	27.4 (13.0)	−5.09	0.410	0.110
AST (iu/L)	19.7 (4.2)	26.6 (11.5)	44.50%	0.320	22.6 (5.2)	21.7 (7.0)	−3.9	0.520	0.200

BMI = Body mass index; WC = waist circumference; WHR = waist/hip ratio; FAI = free androgen index; DHEAS = dehydroepiandrosterone sulphate; SHBG = sex-hormone-binding globulin; LH = luteinizing hormone; FSH = follicle-stimulating hormone; OGTT= oral glucose tolerance test; TG = triglycerides; CRP = C-reactive protein; ALT = alanine aminotransferase; AST = aspartate aminotransferase. Results are expressed as mean (±SD) or percentages. Significant *p*-values are indicated in bold. ^1^ *p*-value for pre-post changes within group. ^2^ *p*-value for difference in % changes between groups.

**Table 3:** Changes in parameters of body composition in women with PCOS with VLCD and moderate energy deficit at 8-week follow-up.

VLCD Arm (*n* = 11)					Moderate Energy Deficit Arm (*n* = 11)				
	Baseline	Follow Up	Mean % Change	*p*-Value ^1^	Baseline	Follow Up	Mean % Change	*p*-Value ^1^	*p*-Value ^2^
Total fat (kg)	53.63 (±86.13)	45.24 (±80.99)	−15.76	**<0.0001**	52.87 (±12.62)	50.49 (±12.99)	−4.91	**0.003**	**<0.0001**
Trunk fat (kg)	28.40 (±40.93)	23.45 (±32.71)	−17.30	**<0.0001**	27.74 (±75.23)	26.49 (±79.91)	−5.16	**0.070**	**<0.0001**
Lean body mass (kg)	50.14 (±49.80)	47.36 (±54.49)	−5.65	**<0.0001**	51.69 (±85.25)	50.65 (±74.19)	−1.78	**0.050**	**0.002**
Fat free mass (kg)	52.83 (±51.57)	50.052 (±55.99)	−5.35	**<0.0001**	53.88 (±73.25)	52.94 (±65.14)	−1.58	**0.050**	**0.001**
BMD	1.28 (±0.12)	1.27 (±0.11)	−0.01	0.060	1.24 (±0.08)	1.25 (±0.11)	0.53	0.720	0.320
BMC	2688.091 (±315.8)	2687.9 (±284.1)	12.00	0.990	2706.45 (309.5)	2697.9 (269.2)	−0.14	0.780	0.830

BMD = bone mineral density, BMC = bone mineral content. Results are expressed as mean (±SD) or percentages. Significant *p*-values are indicated in bold. ^1^ *p*-value for pre-post changes within group. ^2^ *p*-value for difference in % changes between groups.

## Data Availability

The data presented in this study are available on reasonable request from the corresponding author.

## Supplementary Materials

The following supporting information can be downloaded at: https://www.mdpi.com/article/10.3390/nu15183872/s1, Table S1: Summary of data collected at each visit.
