# Supplementary material for: The Effect of a Very-Low-Calorie Diet (VLCD) vs. a Moderate Energy Deficit Diet in Obese Women with Polycystic Ovary Syndrome (PCOS)—A Randomised Controlled Trial
Source: Nutrients. 2023 Sep 6;15(18):3872. doi: 10.3390/nu15183872 (PMC10536436; doi:10.3390/nu15183872)
Supplement: Supplementary file 1 [file nutrients-15-03872-s001.zip › nutrients-2542827-supplementary.pdf]

Table S1. Summary of data collected at each visit.

	**1**	**2**	**3**	**4**	**5**	**6**	**7**	**8**	**9**
Week	1	2	4	6	8	10	12	14	16
Randomisation	x								
Screen and consent to the study	x								
Modified polycystic ovary syndrome health-related quality-of-life questionnaire (MPCOSQ) and Hospital Anxiety and Depression Scale (HADS)		X				X			
Motivation assessment		X	X	X	X				
Weight (kg) and BMI calculation		X				X	X	X	X
Blood Pressure		X				X			
Height (m)	x								
DEXA		X				X			
Endopat 2000		X				X			
Waist circumference and Hip circumference		X				X			
Weight maintenance support						X	X	X	X
Dietetic support ideally via visit (or email, phone, text)		X	x	X	x				
Measure of compliance				X		X			
Collection of drinks sachets (or as required)		X		X		X			
Oral glucose tolerance test		X				X			
Fasting blood tests		X				X			
